# The Inebriates Act

**Published:** 1899-07-08

**Authors:** 


### THE INEBRIATES ACT.

From the recently-presented report of the Special Committee of the London County Council on the Inebriates Act, 1898, we gather that at present there are only three certified reformatories, viz.: (1) The Royal Victoria Homes, Brentry, near Bristol, which propose that the County Council should enter into an agreement (*a*) for not fewer than five cases and for not less than five years; (*b*) to pay £10 per bed per year for capital outlay; (*c*) to pay 6d. per day for each case sent. (2) Lady Somerset's Home, which proposes that the Council should purchase a portion of the freehold and should erect all necessary buildings and pay 16s. a week for each case sent. (3) St. Joseph's Inebriate Reformatory, Ashford, which is for Roman Catholic women. The payment here would be an establishment contribution of £10 per annum for each person, and 3s. 6d. per week each for maintenance. A proposal is also made to establish a reformatory at the Training College at Lingfield, Surrey, to which County Councils would be asked to contribute. After considering the alternatives, the opinion of the committee is in favour of the Council itself establishing a reformatory, rather than joining other county councils or the managers of private institutions for the purpose. In the meantime they suggest that a temporary arrangement, say for twelve months, should be made with one or other of the existing reformatories to meet the cases which may arise in the immediate future, and, acting upon communications which have been received from Lady Henry Somerset and the Superior of the St. Joseph's Inebriate Reformatory, the committee submit to the Finance Committee an estimate of £2,000, which, in view of the Government grant of 10s. 6d. per week for each inmate, it is hoped will cover the cost to the Council of about 100 persons for twelve months.

